# Coffee Somatic Embryogenesis: How Did Research, Experience Gained and Innovations Promote the Commercial Propagation of Elite Clones From the Two Cultivated Species?

**DOI:** 10.3389/fpls.2018.01630

**Published:** 2018-11-12

**Authors:** Hervé Etienne, David Breton, Jean-Christophe Breitler, Benoît Bertrand, Eveline Déchamp, Rayan Awada, Pierre Marraccini, Sophie Léran, Edgardo Alpizar, Claudine Campa, Philippe Courtel, Frédéric Georget, Jean-Paul Ducos

**Affiliations:** ^1^CIRAD, UMR IPME, Montpellier, France; ^2^IPME, Université de Montpellier, IRD, CIRAD, Montpellier, France; ^3^Nestlé R&D Center Tours – Plant Science Research Unit, Tours, France; ^4^ECOM, Exportadora Atlantic, Managua, Nicaragua; ^5^IRD, CIRAD, Université de Montpellier, IPME, Montpellier, France

**Keywords:** technology transfer, innovation, somaclonal variation, cuttings, bioreactors

## Abstract

Since the 1990s, somatic embryogenesis (SE) has enabled the propagation of selected varieties, Arabica F1 hybrid and Robusta clones, originating from the two cultivated coffee species, *Coffea arabica* and *Coffea canephora*, respectively. This paper shows how mostly empirical research has led to successful industrial transfers launched in the 2000s in Latin America, Africa, and Asia. Coffee SE can be considered as a model for other woody perennial crops for the following reasons: (i) a high biological efficiency has been demonstrated for propagated varieties at all developmental stages, and (ii) somaclonal variation is understood and mastered thanks to intensive research combining molecular markers and field observations. Coffee SE is also a useful model given the strong economic constraints that are specific to this species. In brief, SE faced four difficulties: (i) the high cost of SE derived plants compared to the cost of seedlings of conventional varieties, (ii) the logistic problems involved in reaching small-scale coffee growers, (iii) the need for certification, and (iv) the lack of solvency among small-scale producers. Nursery activities were professionalized by introducing varietal certification, quality control with regard to horticultural problems and somaclonal variation, and sanitary control for *Xylella fastidiosa*. In addition, different technology transfers were made to ensure worldwide dissemination of improved F1 Arabica hybrids and Robusta clones. Innovations have been decisive for successful scaling-up and reduction of production costs, such as the development of temporary immersion bioreactors for the mass production of pre-germinated embryos, their direct sowing on horticultural soil, and the propagation of rejuvenated SE plants by rooted mini-cuttings. Today, SE is a powerful tool that is widely used in coffee for biotechnological applications including propagation and genetic transformation. Basic research has recently started taking advantage of optimized SE protocols. Based on -omics methodologies, research aims to decipher the molecular events involved in the key developmental switches of coffee SE. In parallel, a high-throughput screening of active molecules on SE appears to be a promising tool to speed-up the optimization of SE protocols.

## Introduction: From the Proof of Concept of Coffee Somatic Embryogenesis to the Mass Propagation of Selected Elite Clones of Arabica and Robusta

Since pioneer studies by Staritsky (1970), Söndhal and Sharp (1977), Pierson et al. (1983), and Dublin (1984), somatic embryogenesis (SE) has been proposed as an alternative way to propagate elite coffee cultivars from the two cultivated coffee species *Coffea arabica* and *C. canephora*. Tissue cultures of these two coffee species exhibited a high embryogenic potential: the first development of embryoid (direct SE) was reported by Staritsky (1970) and high-frequency embryogenesis from leaf explant-derived friable calli (indirect SE) by Söndhal and Sharp (1977). The morphological and histological characterization of both embryogenic and non-embryogenic calli as well as the histological events leading to the embryo regeneration in direct and indirect SE technologies have been described by many authors in detail until recently (Berthouly and Michaux-Ferriere, 1996; Quiroz-Figueroa et al., 2006; Gatica-Arias et al., 2008; Padua et al., 2014; Silva et al., 2014). These pioneer studies in the seventies provided the proof of concept of coffee SE, but at that time, no selected varieties were available to be propagated. In addition, the studies showed that several well-known bottlenecks hindering the development of SE in other crops such as the difficulty to regenerate embryogenic tissues and a strong genotypic effect were very limited in coffee. Hence most research efforts focused very early on the scaling-up of embryo production in liquid medium (Zamarripa et al., 1991a,b).

The allotetraploid *C. arabica* (2*n* = 4× = 44) autogamous species is mainly grown in Latin America (>80% of world production). The species was introduced using a small number of plants, which resulted in very low genetic diversity in the field (Lashermes et al., 2009). These varieties are lineages reproduced via seeds since the species is self-pollinating. From 1990, CIRAD and its public and private partners [CATIE (Tropical Agricultural Research and Teaching Center, Turrialba, Costa Rica), ICAFEs (Central American Coffee Research Institutes), ECOM-trading (ECOM-trading is a leading global commodity merchant and sustainable supply chain management company)] created F1 hybrid varieties of *C. arabica*, based on the crossing of genetically distant American lineages and wild individuals from Ethiopia and Sudan (van der Vossen et al., 2015) to introduce more genetic diversity. These hybrids enabled a 30–60% increase in production (Bertrand et al., 2011) and also improved the aromatic quality (Bertrand et al., 2006). The selection time of these hybrids, which is significantly shorter than that of conventional lines (8 vs. 25 years), is another argument in their favor. After their phenotyping in field conditions, their initial selection is made by family and then by plant. The product of the selection is a hybrid (heterozygous genetic structure) that has to be vegetatively propagated (cloning) to conserve all the characteristics of the selected mother plant. The annual seed requirements for orchard renewal in Latin America are estimated at several hundred million coffee plants. Given the lack of efficient protocols for horticultural propagation by grafting and cuttings, SE was considered to be the most promising tool to insure the large scale dissemination of hybrids.

Robusta (*C. canephora* var. Robusta) is a strictly allogamous species that is traditionally propagated by seeds. Because of uncontrolled pollination, the planting material generated from seeds is highly heterogeneous, and the yields of the majority of trees are poor. The strong competition between industrial crops for profitable land is putting pressure on the cultivation of Robusta. One way to increase the profitability of the crop is to distribute new clones with higher agronomic performances that can be propagated using vegetative methods like grafting or rooted cuttings. In the 1990s, Nestlé R&D-Tours created their “Core Collection” of 55 clones representative of Robusta diversity, and planted them in the field in Ecuador and Thailand (Pétiard et al., 2004). The biomolecular aspects, agricultural performances, biochemical composition, processing performances and sensory (organoleptic) characteristics of the collection were extensively studied, leading to the selection of the most advantageous clones. From this collection, those with added genetic value were propagated via SE to set up multilocation field trials in several coffee producing countries. With the collaboration of National Institutes, the genotypes were validated under local conditions before being mass propagated by SE and distributed to farmers.

In this paper, we show how three major breakthroughs led to scaling-up of SE in coffee for the mass propagation of selected Robusta clones and Arabica hybrids: (1) in the early 1990s, liquid culture media were used for the multiplication of embryogenic cells and for the production of embryos up to the torpedo-stage (Zamarripa et al., 1991a,b; Ducos et al., 1993; Zamarripa, 1993); (2) in 1995, it became possible to pre-germinate embryos up to the cotyledonary-stage using temporary immersion culture in liquid media (Berthouly et al., 1995; Etienne-Barry et al., 1999); (3) in the late 1990s, it was possible to achieve *ex vitro* germination by direct sowing of cotyledonary embryos in the greenhouse (Ducos et al., 1999; Etienne-Barry et al., 1999).

Starting from this progress, pilot processes for large scale propagation were launched for Arabica in 2002 (CIRAD/ECOM alliance, laboratory of Sebaco, Nicaragua) and for Robusta in 2003 (Nestlé R&D Tours, France). In this paper, we describe the background and main steps in coffee SE from research up to industrial applications and summarize the key research results obtained by these two leading groups in coffee SE. Finally, we present current technological and basic research on coffee SE.

## Arabica Mass Propagation Through Somatic Embryogenesis: the Birth of a Commercial Activity

### *C. arabica* SE: A Model for Perennial Plants Under Strong Economic and Social Constraints

Once a variety of coffee has been created by breeders, it needs to be propagated at a large scale, respecting conformity, to be subsequently distributed to growers at a fair price (Bertrand et al., 2012). In Costa Rica, Honduras, Colombia and Brazil, for example, state or cooperative organizations distribute the seeds of varieties with high germination quality and varietal purity ranging between 90 and 95% for a subsidized cost of US $8–15/kg. In other producing countries (Guatemala, El Salvador, Nicaragua, Peru, Mexico, Panama, Bolivia, Jamaica, and Dominican Republic), basic services for variety improvement and/or seed propagation and certification, do not exist. In these countries, coffee growers have no choice but to turn to non-certified seed producers, which increases the risks of losing varietal purity (due to seed mixtures and cross-pollination), germination capacity (highly variable depending on the batch), and possibly subsequent coffee productivity and quality. Given this situation, and also taking into account that the lifespan of a coffee plant is around 20 to 30 years, it is urgent to structure the coffee seed production chain in producing countries since we know that the choice of the variety to be planted by most producers is based on either the arguments of their neighbor or on (often) outdated information provided by coffee institutes, which increasingly lack the means to fulfill their role as advisors to producers.

The propagation of F1 hybrids creates new problems, both commercial and logistical. Indeed, as these varieties are propagated vegetatively (and not by seeds), they must be distributed to small producers in the form of plantlets grown at the lowest possible cost. Four main difficulties limit the massive distribution of such hybrids: (i) the high cost of F1 plantlets, (ii) the fact that small producers have no capital, (iii) fluctuations in the price of coffee, and (iv) logistic problems in reaching small producers, who often live in remote and mountainous areas. Possible ways to overcome these difficulties are presented below.

#### Horticultural and Agronomic Solutions to Reduce the Cost of F1-Plantlets

Several hundred thousand F1 hybrid plants are produced each year using the SE technique (Etienne et al., 2012, 2016; Bobadilla Landey et al., 2013) by a laboratory located in Sébaco (Nicaragua) and managed by the Agritech Company. The price at the laboratory gate is high (US $0.5–0.6), representing the production cost of a 2 month old acclimatized plantlet derived from a somatic embryo, and the plant material is too heterogeneous.

The recent development of propagation by rooted mini-cuttings (Georget et al., 2017) significantly increased both the number (multiplication rate >10–30) and the quality of plantlets derived from SE and subsequently divided the production costs by half (from a mean of US $0.50–0.60 to 0.25–0.30 per plant).

#### Considering the Solvency of Small Producers

When leaving the nursery, a 6 month-old F1 plant propagated by SE and ready to be planted in the field has a set sales price of around US $0.8–1.0 versus US $0.3–0.5 for a seed-derived plantlet. The cost is still too high for the majority of coffee growers. Knowing that between 4,000 and 6,000 plantlets are required per ha, planting F1 clones represents an additional cost of US $2,000–3,600 per ha compared to the cost of setting up a conventional hectare (between US $4,000 and 7,000 per ha).

In fact, due to the improved productivity of these hybrids and their better resilience, it is easy to show that this extra cost can be refunded after three to four harvests (out of 15–20 harvests that represent the life span of a normal coffee plantation).

This problem could also easily be overcome if the banks considered that small coffee growers (who represent the majority of producers) are solvent. However, because coffee prices are extremely volatile, banks often do not trust small producers and enforce usurious agricultural credit rates (between 8 and 14%). Thus, only a limited number of coffee growers (the biggest) are able to take the financial risk of planting F1 hybrids (or even of modernizing their plantations).

#### Fluctuating Coffee Prices and Direct Trading

One way to improve the living conditions of coffee growers and their production tools is to not consider produced coffee as a raw material whose price is fixed on the stock market by speculators, but to consider it as a product with a price that can be fixed between the grower and a buyer (roaster) who will guarantee a fair price and limited fluctuation over a long period (direct trading). More and more traders, notably ECOM are turning to this type of trading. Once the price is fairly established between them, it will be easier for the coffee grower to obtain credit at a reasonable rate.

#### Logistic Problems Involved in Trying to Reach Small Producers and Setting Up Cooperatives

Since the remoteness of many of the coffee growers made industrialization impossible, we developed a concept of rooted mini-cuttings in a rural environment. Inspired by farmers’ seed systems, which are mainly suitable for seed reproduction, networks called “cooperatives” were created. The horticultural technique of rooted mini-cuttings derived from somatic embryo-derived plants (somatic seedlings) was transferred to these women’s cooperatives in order to (i) reduce the production costs of mini-cuttings, (ii) reduce gender inequalities, and (iii) facilitate access to F1 hybrids and their use. This experiment is currently being tested in three very different countries (Vietnam, Cameroon, and Nicaragua) in the framework of the BREEDCAFS H2020 project (BREEDing Coffee for Agroforestry Systems^1^) supported by the European Commission.

From a small number of initial SE-derived plantlets provided by a certified plantlet producer and renewed every year, women cooperatives commit to produce rooted mini-cuttings they then sell to coffee growers. The cooperatives also commit to pay a royalty recognizing the breeder’s right each year.

### Breakthrough Innovations and Research to Scale Up the Technology and Reduce Production Costs

#### Mass Production of Pre-germinated Embryos in Temporary Immersion Bioreactors

**FIGURE 1:**
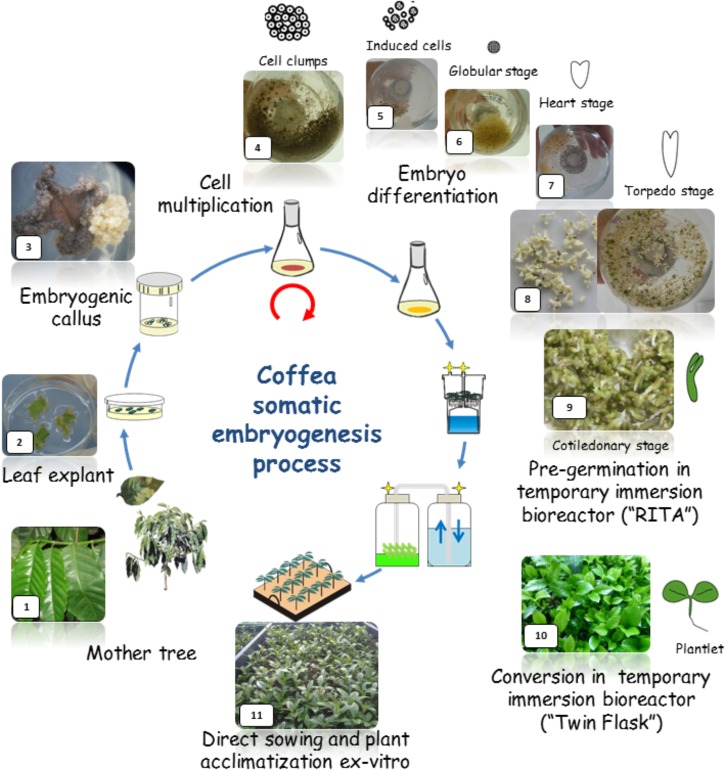
Schematic representation of the *Coffea arabica* mass propagation process (24 months) through somatic embryogenesis (SE). The process is based on the use of liquid nutritive media for the embryogenic tissue multiplication and embryo regeneration phase and for the pre-germinated embryo and plantlet bulk production in temporary bioreactors (1L-RITA, 5L-MATIS and 3L-Twin-flasks). Direct sowing of germinated embryos and plantlets onto horticultural substrate avoids the use of gel media and requires less manpower in *in vitro* conditions.

CIRAD was a pioneer in the development of temporary immersion bioreactors, whose advantages for micropropagation were clearly demonstrated in the coffee SE model through high yielding and reliable processes (Figure 1). Twenty clones of *C. arabica* F1 hybrids successfully reproduced using a 1 L-RITA^®^ temporary immersion bioreactor (Etienne et al., 1997). Depending on the genotype, yields ranged from 15,000 to 50,000 somatic embryos per gram of embryogenic suspension. The most spectacular effect obtained by temporary immersion was a dramatic improvement in the quality of the resulting somatic embryos. While the observed proportion of normal torpedo-shaped embryos is usually 20–30% in a bioreactor or in Erlenmeyer flasks (Zamarripa et al., 1991a; Noriega and Söndhal, 1993; De Feria et al., 2003), with temporary immersion, the proportion reached over 90%. This improvement in quality is also reflected in higher plant conversion rates on agar medium (80–90%) and, more particularly, in the highly successful regeneration of plants after direct sowing on horticultural substrate (Barry-Etienne et al., 1999). The adjustment of immersion time is critical with this type of bioreactor. According to Teisson and Alvard (1995) with 1-L bioreactors, 15 min of immersion at 6 h intervals led to repetitive embryogenesis and germination of coffee embryos, whereas in an identical culture medium, 1 min immersions at 24 h intervals halted embryo development and stimulated the production of adventive embryos. The use of bigger temporary immersion bioreactors, like the new 5 liter (5L)-MATIS^®^ (area 355 cm^2^) bioreactor designed horizontally specifically to favor embryo dispersion and light transmittance to somatic embryos, further improved (91%) embryo-to-plantlet conversion, for the first time, enabling the regeneration of large numbers of Arabica plantlets in a bioreactor (Etienne et al., 2013). Similarly, protocols for bulk mass production of pre-germinated embryos in temporary immersion bioreactors were also developed with the other crop species *C. canephora* (Ducos et al., 2007a,b).

#### Direct Sowing in a Nursery of Pre-germinated Embryos

To further reduce production costs, the plant conversion steps on gel media in the laboratory had to be eliminated because they require a lot of labor for transplanting, many culture containers to be used and washed and a lot of shelf space. The effect of the germination conditions on the morphology of *C. arabica* somatic embryos mass-produced in a 1 L bioreactor was shown to play a determining role in successful plant conversion in the nursery after direct sowing in soil (Figure 1). Using pre-germinated embryos, direct sowing resulted in a highly successful conversion of embryos into plantlets. A culture density of more than 1,600 embryos per 1 L-bioreactor affected embryo morphology positively as it caused higher embryonic axis elongation (+4 to 5 mm) (Barry-Etienne et al., 1999). Furthermore, direct sowing reduced handling time to 13% and the required shelf space to 6.3% of those required by conventional acclimatization of plants developed on gel media.

In this way, approximately 800 Arabica somatic embryos were obtained in a 1L-RITA^®^ bioreactor, 86% of which reached the pre-germinated stage but with morphological heterogeneity (Barry-Etienne et al., 2002a). For example, the pre-germinated embryos can be sub-divided into three categories according to cotyledon area which is proportional to embryo age: “small” (32%), “medium” (36%), and “large” (4.5%), the remaining material comprising non-germinated embryos. Embryos with an intermediate (medium) cotyledon area (0.86 cm^2^) were shown to not only have the best plant conversion rates *ex vitro* (63%), but also resulted in vigorous plantlets. Compared to *in vitro* germination conditions, mortality was higher in nursery conditions, but better plant development was obtained (Barry-Etienne et al., 2002b). The quality of plantlets produced in *ex vitro* conditions was reflected in the better growth of the aerial and root systems, but also by similar morphological, mineral and water status characteristics to those of seedlings. Direct sowing of pre-germinated somatic embryos therefore rapidly resulted in vigorous plantlets in *ex vitro* conditions, whilst eliminating the need for tricky and costly conventional acclimatization procedures. However, it was also clearly demonstrated that the heterogeneity of the embryos in the bioreactor resulted in variability in both the plant conversion efficiency in soil and plant growth in the nursery, delaying growth, mainly in plantlets derived from somatic embryos with small cotyledons (Barry-Etienne et al., 2002a).

#### Rooted Mini-Cuttings From Somatic Seedlings

**FIGURE 2:**
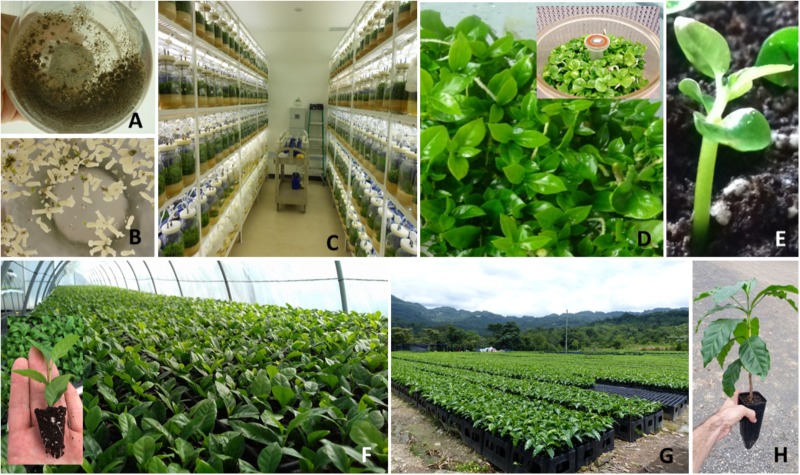
Somatic embryogenesis of *C. arabica*: model of industrialization and professionalization. **(A)** Embryogenic cell suspension in liquid medium; **(B)** embryo differentiation in liquid medium (*torpedo stage*); **(C)** embryo development phase in TIS (temporary immersion system); **(D)** germinated embryos in Twin-Flask Bioreactor (above: cotyledonary embryos obtained in 1L-RITA); **(E)** Conversion into plantlets of pregerminated embryos onto horticultural substrate; **(F)** rooted cuttings in plug trays; **(G)** industrial hybrid nurseries with plastic pots and peat moss substrate. **(H)** Rooted cutting ready for field planting.

Based on these facts, work was done to reduce the nursery plant heterogeneity and production costs. Mature tissues of *C. arabica* are known to be recalcitrant to rooting (around 8–12% rooting efficiency). However, we observed that between 10 and 25 weeks of development in the nursery, SE plantlets derived from *C. arabica* hybrids were temporarily able to root with a high success rate (up to 90%) whatever the genotype tested, before gradually losing that capacity. We took advantage of this transient rooting capacity, which was probably due to the rejuvenation process occurring during SE (resulting from the successive cell dedifferentiation/re-differentiation mechanisms), to establish a new propagation system in nursery conditions based on the serial rooting of cuttings obtained from an initial SE-derived plant batch, known as horticultural rooted mini-cutting (HRMC) (Georget et al., 2017). The excessively low embryo-to-plantlet conversion rates (around 50%) during acclimatization and hardening steps in nursery that limit SE profitability can be more or less offset by the much higher HRMC multiplication rate (14 in 6 months) and better overall quality (Figure 2). Fifteen week-old rooted mini-cuttings were more uniform (2–4.5 cm vs. 1–5.5 cm for plant height distribution) and vigorous (1.41 vs. 0.81 mm for stem diameter) than SE-derived plantlets of the same age. We showed that this effect persists for several years after planting. Moreover, SV were not observed in the plants propagated by HRMC.

#### Long-Term Studies Led to the Control of Somaclonal Variation

**FIGURE 3:**
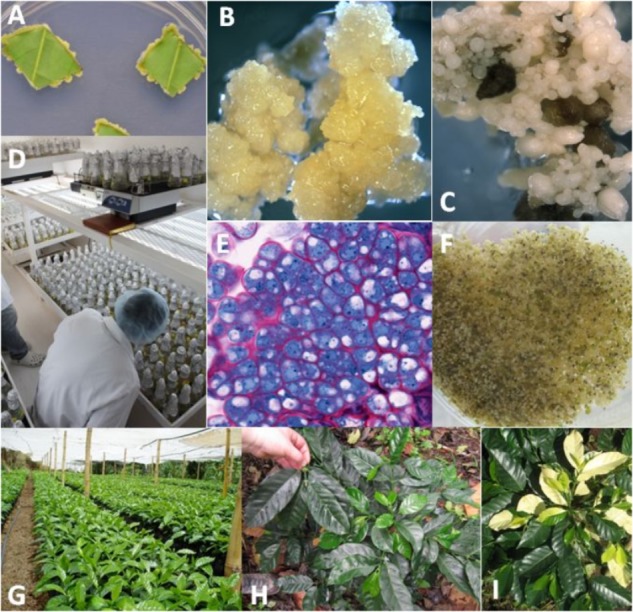
Illustration of the production, multiplication, and plant regeneration from embryogenic cells in coffee (*C. arabica*). **(A)** Formation of a primary callus made of meristematic cells at the edge of leaf explants 3 weeks following *in vitro* introduction; **(B)** embryogenic callus obtained 6 months after *in vitro* introduction; **(C)** regeneration of somatic embryos from the embryogenic callus; **(D)** proliferation of embryogenic cell suspensions and subsequent regeneration of somatic embryos in Erlenmeyer flasks agitated on orbital shakers in an industrial coffee micropropagation laboratory (CIRAD/ECOM unit, Sebaco, Nicaragua); **(E)** histological appearance of embryogenic cell clusters in suspension cultures; embryogenic cells have a high nucleus cytoplasm ratio and dense cytoplasm rich in soluble and reserve proteins; **(F)** mass regeneration of torpedo-stage somatic embryos from embryogenic cell suspensions; **(G)** coffee SE-derived plants in the nursery (Cartago, Costa Rica); **(H)** “Dwarf” phenotypic variant (on the right) compared to a normal plant on the left; **(I)** “Variegata” phenotypic variant *(from Etienne et al., 2016).*

To be able to use industrial scale procedures, developing embryogenic cell suspensions is the best way to ensure synchronous and massive somatic embryo production, and this process can be easily scaled up in bioreactors. Although embryogenic cell suspensions have been developed for some major crops, this has not been the case for commercial uses. The use of embryogenic cell suspensions has frequently been associated with the increased likelihood of genetic and epigenetic instability and somaclonal variation (SV) in the regenerated plants (Etienne et al., 2016). Preliminary studies on coffee showed that seven types of phenotypic variants [Color of juvenile leaves, Giant, Dwarf, Thick leaf (Bullata), Variegata, Angustifolia, and Multi-stem] were systematically observed in all the regenerated plant batches (Etienne and Bertrand, 2001, 2003; Figure 3). For all genotypes, the degree of SV was always low (around 1.3%) for plants directly produced from embryogenic callus or 3 month old embryogenic cell suspensions. Thereafter, the frequency of variants increased significantly, reaching 6, 10, and 25% in plants produced from suspensions aged 6, 9, and 12 months, respectively. It was also observed that the severity of SV increased with the age of the embryogenic suspension with the successive appearance of mainly of “Dwarf,” “Angustifolia,” and “Multi-stem” phenotypic variants.

We conducted further research into industrial culture conditions, which were expected to be weakly mutagenic thanks to the combined use of a short proliferation period (6 months) and low auxin supply (0–1.4 μM 2,4-D) and compared two proliferation systems: secondary embryogenesis and embryogenic cell suspensions (Bobadilla Landey et al., 2013). Independently of the proliferation system studied, amplified fragment length polymorphism (AFLP) and methylation-sensitive amplified polymorphism (MSAP) molecular analyses on 145 SE derived-plants showed that genetic and epigenetic polymorphisms between mother plants and SE derived-plants were always low but present, ranging from 0 to 0.003% and 0.07 to 0.18%, respectively. Based on MSAP analyses, no plant was found to cumulate more than three methylation polymorphisms. It is expected that more powerful molecular tools could evidence more variations. Moreover, *C. arabica* is a young allopolyploid still having the most of its genes in duplicated copies (Cenci et al., 2012). It could be hypothesized that the impact of genetic or epigenetic variations on phenotype was restricted because of the buffer effect due to polyploidy.

Massive phenotypic observations in the nursery and field plots revealed very low levels of SV (0.9% in 800,000 plants), probably due to the short duration of the proliferation period applied as cell culture aging has clearly proved to strongly enhance SV (Etienne and Bertrand, 2003; Bobadilla Landey et al., 2015). Cytological analyses showed that most coffee somaclonal variants presented abnormal chromosome numbers (41–43, 45) instead of 44 as in phenotypically normal plants of *C. arabica*. The presence of mitotic aberrations, including double prophase, lagging chromosomes, aneuploid and polyploid cells, has previously been described in leaves (Ménendez Yuffá et al., 2000; Zoriniants et al., 2003) and embryogenic calli (Ménendez Yuffá et al., 2000) of *C. arabica* but not in the later steps of SE, and without establishing any relation with SV.

Stressful experimental conditions were also applied by using extended proliferation periods (4, 12, and 27 months) for three independent embryogenic cell lines established in the presence of high concentrations of growth regulators (4.5 μM 2,4-D, 17.8 μM 6-benzylaminopurine [6-BA]) to understand the mechanisms of culture aging and their relations with SV (Bobadilla Landey et al., 2015). This showed that the proliferation time strongly affected the SV frequency in the regenerated plants (*n* = 180) and in a very similar way in the three embryogenic cell lines. No variants were found after 4 months of proliferation whereas 30 and 94% phenotypic variants were observed in plants derived from 12 and 27 month cultures, respectively. Like the plants produced in industrial conditions, phenotypic variants systematically showed abnormal chromosome numbers.

Our research thus clearly demonstrated that SE based on embryogenic cell suspensions cultured for less than 6 months with low auxin supply was efficient and reliable for true-to-type propagation of selected *C. arabica* varieties with over 99% of regenerated coffee trees in full conformity with the mother plant morphologically and conserved their original physiological and agronomic traits.

### Arabica Somatic Embryogenesis: From Pilot Scale to Commercial Production

#### Scaling-Up Arabica Somatic Embryogenesis

Between 1995 and 2001, CIRAD, in partnership with the coffee institutes of Central America (ICAFE-Costa Rica, IHCAFE-Honduras, PROCAFE-Salvador, ANACAFE-Guatemala) and CATIE (Costa Rica), conducted laboratory research that led to mass production in bioreactors and the possibility of sowing in the nursery. Over this period, tens of thousands of plants were produced every year. In the meantime, reassuring data were obtained concerning the low rate of SV and the better performances of F1 hybrids compared to pure line varieties, especially their remarkable adaptation to both full sun and agroforestry conditions (Bertrand et al., 2011).

In 2002, sufficient information had been collected to test F1 hybrids and SE on a larger scale. In partnership with a private company (ECOM trading group), CIRAD decided to apply the SE method to four *C. arabica* F1 clones first at the pilot scale, and subsequently at industrial scale in Nicaragua. The first commercial production of vitroplants took place in 2008. Between 2008 and 2010, more than 5 million pre-germinated embryos were produced in the laboratory and nearly 2 M hybrid plants were distributed to coffee growers mainly located in Nicaragua (Etienne et al., 2012). Over this period, several technical advances in the SE process helped support this change in scale. The first was the mastery of embryogenic suspensions and the production of somatic embryos in liquid medium in Erlenmeyer flasks. This innovation resulted in a dramatic reduction in production costs associated with the cost of labor, and also contributed to the synchronization and homogenization of plant material development compared to the original protocol – asynchronous repetitive SE in RITA^®^ bioreactors. Two distinct developmental stages were characterized, “cell aggregates” in the multiplication phase (Figure 2A) and torpedo-shaped embryos at the end of the differentiation phase (Figure 2B). The highly embryogenic potential of Arabica cell suspensions was also reported (e.g., 250,000–500,000 embryos/mL of cell suspension).

In 2010, *C. arabica* SE was relatively well mastered at the semi-industrial level (Etienne et al., 2012; Bobadilla Landey et al., 2013), but some technical constraints remained that prevented its scaling-up: a low embryo-to-plantlet conversion rate obtained in RITA (50%) associated with a long acclimatization stage (between 20 and 30 weeks) and obvious asynchronicity (2 to 3 harvests were needed to collect all the plantlets derived from embryo sowing). However, major optimizations were achieved over the period 2011 to 2018, which allowed the production of hybrids to be scaled up. Among these optimizations was the use of large Twin-Flask bioreactors (Figure 2C) leading to an increased plant conversion rate after sowing (Figures 2D,E) (70%) and the development of rooted mini-cuttings (Figure 2F). These mini-cuttings made it possible to use the same horticultural methodologies for coffee plants as those already available for industrial production of other crops (automated filling of the containers, watering and ferti-irrigation, automatic climate management, manufacturing of customized horticultural containers, etc.) (Figures 2G,H). The result was a gain in reproducibility enabling the production of high quality SE derived-plants at a lower cost.

In the laboratory full traceability of production was also achieved with the systematic verification of mother plants using the SNP microsatellite markers previously used in our laboratory (Combes et al., 2000). Similarly, to prevent the risk of a health threat linked to the presence of *Xylella fastidiosa*, mother plants, as well as some progeny plants, are systematically indexed using a commercial detection kit^2^ to prevent the risk to plant health linked to the presence of *X. fastidiosa* reported to be detected in coffee plants elsewhere (Jacques et al., 2016).

Regarding SV, even though it was demonstrated that the new SE industrial protocol generated very few variants (see above) a strategy for risk dilution was also created (Etienne et al., 2016). It consists in (i) introducing full production traceability through the creation of lots and production lines (plants derived from the same embryogenic calli), (ii) limiting the number of plants produced per line (100,000 plants) and the cell suspension age to 6 months, and (iii) early screening of somaclonal variants in nurseries, given that most coffee mutants are phenotypically detectable at this stage. These different measures allowed us to routinely obtain mutation rates of less than 0.5%. Today, the CIRAD/ECOM alliance produces between 2 and 3 M hybrid plants per year in Nicaragua, and regularly exports several hundred thousand to neighboring countries (El Salvador, Mexico, Guatemala, Costa Rica, and Peru).

#### The Professionalization of the Production of Arabica Hybrid Vitroplants

In the absence of private seed companies in the coffee business, one of our main objectives was to professionalize the production of hybrid vitroplants of *C. arabica* to ensure their distribution on a very large scale (several hundred million), with full traceability, excellent horticultural and phytosanitary quality (i.e., disease and pest free) and following a well-defined and innovative technical itinerary. In recent years, the CIRAD/ECOM alliance has initiated this process in Nicaragua with the help of the World Coffee Research (WCR), which supported the verification and certification program for varieties and producing nurseries. WCR Verified? is the first global standard to confirm that producers of SE-derived F1 hybrids and associated nurseries produce healthy and genetically pure plants^3^. A catalog of WCR-recommended varieties was published in 2017, including the new F1 hybrid varieties jointly developed by CIRAD/ECOM, which represents a first step in the restructuring of the coffee seed sector^4^. Finally, ECOM is currently registering the new hybrid varieties in the UPOV (International Union for the Protection of New Varieties of Plants) in order to recognize breeders’ selection work, limit free access and consequently better regulate their propagation.

### Technology Transfers and Somatic Embryogenesis Democratization for the Propagation of Selected Arabica Hybrids

#### Industrial Prototype in Nicaragua

**FIGURE 4:**
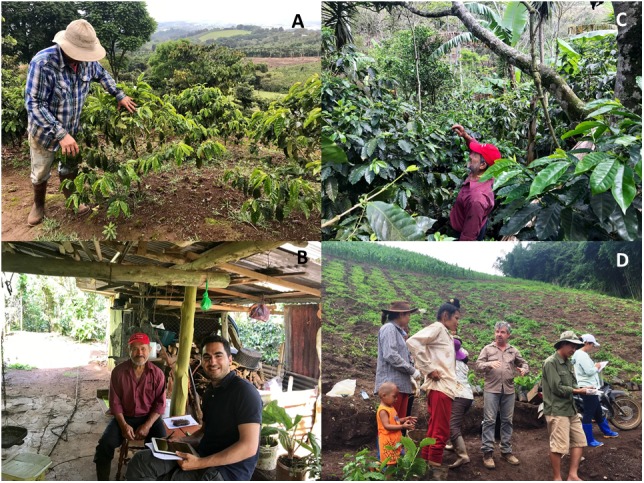
Promoting participatory research and assistance to farmers with new hybrid plant material. **(A)** Farmer showing “his” Arabica hybrid plants in a farm located in Naranjo at 1300 m a.s.l., Alajuela province, Costa Rica; **(B,C)** visit of a farmer in San Ramon (1050 m a.s.l.), Alajuela province, Costa Rica; **(D)** planting of Arabica hybrids in “on farm” evaluation trials in the Sõn La province, Vietnam.

The CIRAD-ECOM alliance promotes participatory research and assists farmers with this new hybrid plant material (Figure 4), which is a complementary activity to that of SE technology transfer. This collaboration led to the construction of an *in vitro* production unit in Sebacco (Nicaragua) and associated industrial nurseries in Matagalpa (Nicaragua) and Cartago (Costa Rica) that are still active today. In the last 10 years, more than 20 million Arabica hybrid plants have been planted in Central America.

#### Democratization of Arabica SE and Rooted Mini-Cuttings (Cameroon, Vietnam, Nicaragua)

**FIGURE 5:**
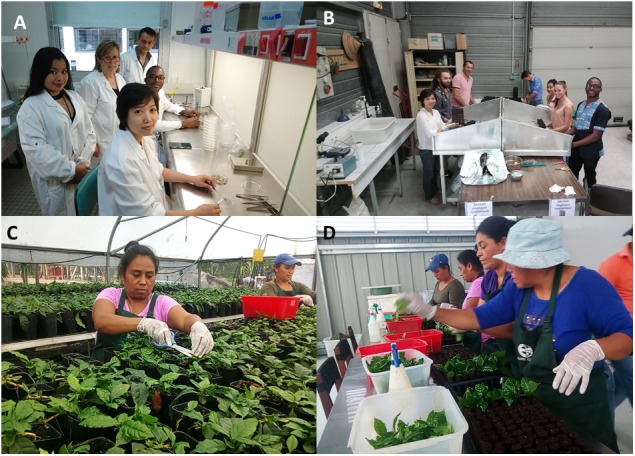
Technology transfer of *C. arabica* SE and democratization of the rooted mini-cuttings companion method. **(A,B)** Training in SE at CIRAD (France) for researchers from Cameroon and Vietnam. **(C,D)** Women’s mini-cuttings cooperative in Nicaragua at the “La Cumplida” farm, Matagalpa district.

Dissemination procedures must include an economic perspective and prevent monopolistic practices and exclusive control of new genetic material. The Mesoamerican experience paves the way for mass production and easy dissemination to farmers of millions of rooted mini-cuttings derived from SE (Georget et al., 2017). The objective of the BREEDCAFS European project (2017–2021) is to popularize these methods in Asia, Africa, and Latin America to allow the mass propagation of hybrids and the diversification of the actors involved in this propagation. In this context, procedure handbooks were distributed to partners, and theoretical and practical courses in technology transfer of SE started in 2017 for personnel from IRAD (Institute of Agricultural Research for Development, Cameroon) and AGI (Agricultural Genetics Institute, Vietnam) (Figures 5A,B).

The role of women in coffee production is often considered minor, despite the fact that they are traditionally responsible for seed production in micropropagation laboratories and micro-cuttings/micro-grafting nurseries. Our objective is to promote the setting-up of women’s mini-cuttings cooperatives. In Cameroon, transfer of the technology is underway through COPAFERLOS, a cooperative of women from the coastal, west and south-west regions, which plays an important role in the education and promotion of women in agriculture. Women’s mini-cutting cooperatives have been created in Nicaragua in the Matagalpa district (“La Cumplida” farm, Figures 5C,D) and in the north western provinces of Sõn La and 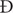
iėn Biên Ph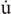 in Vietnam. The goal of the project is to assess the impact of these cooperatives on women, particularly in improving access to, and control of, benefits derived from project participation. Innovation platforms have also been set up to promote dialog among stakeholders of the coffee value chain with the goal of facilitating the acceptance and efficient insertion of both propagation by SE/mini-cuttings and Arabica F1 hybrids in local economies.

## Mass Propagation of Robusta by Somatic Embryogenesis

Since three decades, the Nestlé R&D Center Tours – Plant Science Research Unit has developed intensive research on SE and technological transfers to ensure the large scale dissemination of improved Robusta clones.

### Robusta SE in Liquid Medium: Highlighting a Remarkable Mass Production Potential

#### Multiplication of the Embryogenic Callus

For the two Arabica and Canephora (cv. Robusta) cultivated species, both direct and indirect SE processes have been established showing better performances in the latter species. Direct SE allows the quick embryo regeneration (4 months after *in vitro* introduction) directly on the leaf explants without visible embryogenic callus production. However, as this process is highly asynchronous and leads to the production of low embryo quantities, its use was very early limited to basic studies on cell dedifferentiation/redifferentiation through histological approaches (Berthouly and Michaux-Ferriere, 1996; Quiroz-Figueroa et al., 2006), gene expression (Arroyo-Herrera et al., 2008), epigenetic regulations (Nic-Can et al., 2013), proteomics (Mukul-López et al., 2012), or to the establishment of *in vitro* germplasm core collections. Conversely, indirect SE was investigated in order to establish mass propagation protocols based on the addition of an embryogenic callus multiplication step, starting with *C. canephora*, the less recalcitrant species. These protocols had to be further adapted for Arabica.

**FIGURE 6:**
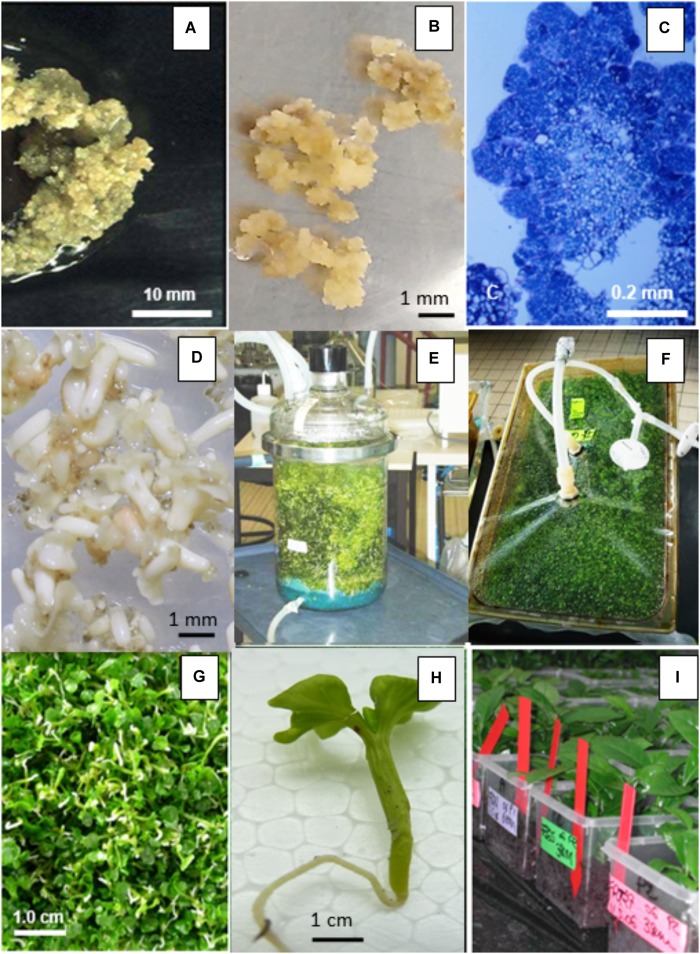
Robusta somatic embryogenic tissues at different stages. **(A)** Primary embryogenic callus on a leaf explant, **(B)** embryogenic calli, **(C)** histological view of an embryogenic clump, **(D)** expression: torpedo-stage embryos at the end of the phase, **(E)** pre-germination: 10 L glass temporary immersion bioreactor (upper recipient), **(F)** Pre-germination: 10 L Box-in-Bag disposable bioreactor (upper recipient), **(G)** pre-germination: aspect of the cotyledonary-stage embryos, **(H)** selected cotyledonary embryos (pre-germinated embryos), **(I)** *ex vitro* germination test in the Nestlé R&D Tours greenhouse.

The multiplication of embryogenic cells in liquid medium is a key step because it greatly and rapidly scales up the number of potential embryos that can be produced. The first establishment and growth kinetics of a coffee cell line were reported in the early 1990s for a model Robusta clone (R2) of *C. canephora* (Zamarripa, 1993). R2 embryogenic callus were induced and multiplied in Yasuda et al. (1985) medium containing 6-benzylaminopurine (BA) at 1.0 mg L^-1^ as the only growth hormone. The R2 cell line was cultured in 0.25-L Erlenmeyer flasks starting with an initial cell density of 10 g FW L^-1^. Growth occurred without an exponential phase and a maximum cell density of 60 g FW L^-1^ was reached after 45 days when the carbohydrates were consumed. This cell line consisted of clumps ranging from 500 to 2,000 μm in size, composed of two cell types (Figures 6A,B). In the center of the clumps, the cells were vacuolated and highly loaded with voluminous starch grains. At the periphery, cells presented a high nucleoplasmic ratio, a slightly vacuolated cytoplasm containing small starch granules and frequent mitotic figures (Figure 6C). These cells, also characterized in Arabica embryogenic cell suspensions, which can be defined as “embryogenic potential” cells, were clustered in peripheral nodules and gave rise to new nodules via their mitotic activity.

#### Expression Step

The production of R2 embryos in 0.25 L flasks was induced by transferring embryogenic clumps to a fresh Dublin (1984) medium consisting of macro and micro MS salts supplemented with 5.0 mg L^-1^ BA, 40 mg L^-1^ adenine, and 400 mg L^-1^ malt extract. The initial cell density was identified as the key factor in causing the cell suspension to produce embryos, showing that low densities of 0.5–1.0 g FW L^-1^ were optimal. After 6 weeks of culture, the total embryo concentration reached a plateau as high as 240,000 L^-1^ with 40% of the embryos at the torpedo-stage (Figure 6D; Zamarripa et al., 1991a,b). On the contrary, high inoculation densities strongly inhibited SE. The same observation is true for the development of SE, expressed by the percentage of torpedo-stage embryos: the lower the initial density, the better their development. Similarly, inoculation density has shown to be a determinant factor for the regeneration of Arabica suspensions. Weekly renewal of the medium partly eliminated this inhibition. The inhibiting activity of the medium conditioned by cells cultured under high cell densities was related to low molecular weight factor(s), below 1 kDa, having a hydrophobic characteristic (Ducos et al., 2007c). After 6 to 8 weeks, the embryos were collected and cultured on solid medium containing 0.2 mg L^-1^ BA for 4 weeks during which they turned green and acquired well-developed cotyledons. The germination steps were conducted *in vitro* by transferring the somatic embryos onto a similar medium, but without BA. With this two-media sequence, up to 70% of the embryos developed into plantlets bearing two to three pairs of leaves. When these plantlets were acclimatized in the greenhouse, the survival rate was more than 90%.

In a bioreactor, critical parameters for the success of mass regeneration of coffee embryos were investigated using a mechanically stirred fermentation apparatus operating at a 3 L working volume (Setric SGI, model SET4CV) (Ducos et al., 1993, 2007c). Although the agitation was provided by a 4-blade “cell-lift” propeller to minimize the shear stress, an initial agitation of 100 rpm had a detrimental effect on growth. Consequently, agitation was kept at the lowest level (50 rpm) until day 21 and then gradually increased to 100–120 rpm. The optimal initial oxygen transfer rate (kLa) was 5 h^-1^. The bioreactor was charged with R2 embryogenic cells at a rate of 0.5 g FW L^-1^. A maximum production of 180,000 embryos L^-1^ was reached on day 58. The first torpedo-shaped embryos appeared on day 28 and 70% of the embryos had reached this stage at the end of the culture.

Mass production of *C. arabica* SE in a 5-L stirred bioreactor were reported by Noriega and Söndhal (1993). A total yield of 45,000 embryos were recovered within 3 months. De Feria et al. (2003) produced *C. arabica* SE in 2-L bioreactors with a rate of 70,000 embryo per g FW of inoculated calli. Beside the facility of the scaling-up of the volume which reduces the labor workforce, another advantage of the bioreactors is the possibility they offer to control various parameters as the aeration rate and the gas composition in CO_2_, ethylene (C_2_H_4_) and particularly in oxygen dissolved concentration (DO_2_). Various works reported the importance of aeration in coffee SE in stirred bioreactor. De Feria et al. (2003) showed that high DO_2_ induced globular SE differentiation in Arabica, but for production of torpedo-shaped SE lower concentrations DO_2_ (<50%) are needed. In the same species, Barbón et al. (2008) reported that the ventilation with an air mixture enriched with CO_2_ at 5% led to more embryos than the control without CO_2_ enrichment. In cyclamen, the regeneration ability of cell suspensions after being cultured in bioreactors with CO_2_ accumulation was better than those after culture in bioreactors without CO_2_ accumulation (Hohe et al., 1999).

#### Application to Selected Clones From the Core Collection

It was confirmed that auxins were not required to obtain embryogenic calli and to multiply them in the majority of Robusta genotypes. Contrary to Arabica, these steps could be managed using a medium containing only a cytokinin as plant growth regulator. However, for some genotypes, even if direct SE occurs during induction, the quantity of embryogenic callus is insufficient and, for the first 5 months, the foliar explants need to be cultured on Pierson medium (Pierson et al., 1983), containing indole butyric acid (IBA) at 5 mg L^-1^.

**FIGURE 7:**
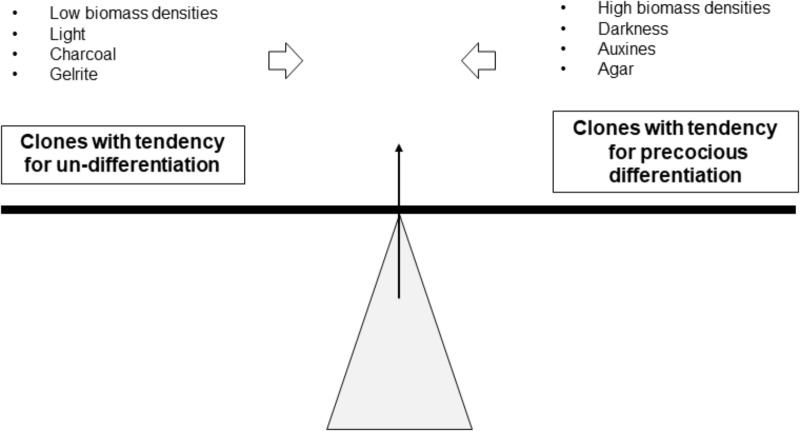
Notion of the “balance” and the effects of some factors on reaching the required equilibrium for the formation of embryogenic callus.

At each subculture, the callus should be carefully selected to maintain a callus stock available on solid medium. This is a repetitive job that requires well trained operators and whose difficulty can be illustrated by the notion of “balance” (Figure 7). The ideal situation is an “equilibrium” between the undifferentiated status of the callus and their embryogenic potential. Typically, some genotypes “lean” to the left, others to the right. Agar, darkness, auxin, and high densities can help to limit precocious differentiation. Inversely, Gelrite, light and low densities facilitate differentiation.

To establish the cell line, a protocol was validated according to which the culture volumes are progressively increased by maintaining high concentrations of biomass, to avoid precocious differentiation, and by keeping the ratio of the volume medium to the volume of recipient identical throughout (Ducos et al., 2007b). At T0, 0.1 g friable callus is transferred into 10 mL of liquid medium in 25 mL flasks. After 2 weeks, the medium is discarded and the tissues transferred into 20 mL of fresh medium in 50 mL flasks. This is repeated every 2 weeks: 50 mL in 100 ml flasks at 4 week, 100 mL in 250 mL flasks at 6 week. In this way, each cell line is started with 0.1 g of selected callus and will produce 1.0 to 2.0 g of callus after 8 weeks of multiplication in liquid Yasuda medium.

#### True-to-Type Status of the Regenerated Plants

In order to validate Robusta propagation via SE in liquid medium, the true-to-type quality of the regenerated trees was checked in field conditions (Ducos et al., 2003). Field trials were run in five coffee-producing countries: Philippines, Thailand, Mexico, Nigeria, and Brazil from 1996 to 2000. These trials represented a total of 12,000 somatic seedlings from 10 clones. In the Philippines and in Thailand, a total of 5,000 trees of five clones (FRT01, 03, 06, 07, and 103) originating from 5 to 7 month old embryogenic cell lines were compared with control trees derived from *in vitro* axillary budding (microcuttings). No phenotypic variant trees were detected. No significant differences in the morphological traits studied and the characteristic yield between trees regenerated were observed using the two methods. Similar to what has been observed in Arabica, these studies in the Robusta species thus confirmed that this method of propagation preserves the agronomic traits to be applied for large-scale applications.

### Pilot Scale Process for the Production of Pre-germinated Embryos

#### Description of the Process

**FIGURE 8:**
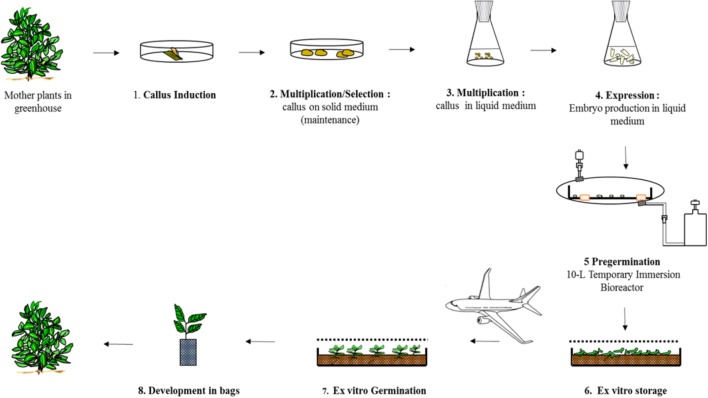
Mass propagation of selected Robusta clones at Nestlé R&D Center Tours.

In 2003, a standard procedure was established to implement the process schematized in Figure 8 at pilot scale (Ducos et al., 2007b). Typically, a batch lasts 6 months, from the initiation of the cell lines up to harvesting the embryos from the bioreactors (steps 3+4+5). Over a period of 3 years, starting from 0.1 g of selected callus from solid medium, we obtained 1.0 kg of callus after multiplication in liquid medium, then 4.4 M pre-germinated embryos. To produce 1.0 M pre-germinated embryos per year, a full time employee was required in the laboratory. Depending on the clone, the embryo-to-plantlet conversion rate varied from 25 to 67%, an average of 41% for 17 clones.

#### Pre-germination in a 10 L Temporary Immersion Bioreactor

To scale up the pre-germination step, we developed a temporary immersion system comprising two glass bottles (a 10 L jar containing the embryos and a 5 L jar containing the culture medium) (Figure 6E). The collected embryo populations differed notably in size, from the early (1 mm) to fully expanded cotyledon stage (20 mm). The only embryos scored as “pre-germinated” are those with an hypocotyl longer than 5 mm which applied to only half the embryos. Light was obviously a limiting factor during culture, as it can only penetrate the first few centimeters of the biomass. We thus hypothesized that a horizontal design would be better than a vertical one as it would provide more light to the embryos thanks to a greater surface-to-volume ratio. The simplest solution consisted in placing a rigid polycarbonate box inside a disposable plastic bag (Figure 6F; Ducos et al., 2007a, 2008). This so-called “Box-in-Bag” bioreactor almost doubled the torpedo-to-pre-germinated-stage conversion rate: for identical inoculum, 16,000 pre-germinated embryos (Figures 6G,H) were collected per bioreactor instead of 8,600 per 10-L glass bioreactor (Ducos et al., 2010).

#### *Ex vitro* Germination in the Greenhouse in a Micro-Environment

Typically, the Robusta embryos are grown on peat or coconut fibers under a 0.5–1 m high plastic tunnel to maintain >95% relative humidity (Figure 6I). However, we observed that simply placing a transparent cover over the containers at a height of 2 to 3 cm above the embryos, significantly increased the *ex vitro* germination rates (Ducos et al., 2009). Due to the micro-environment, the germination rates increased from 42 to 66%. We showed that this positive effect was related to the CO_2_ released by the horticultural media because enrichment with exogenous CO_2_ promoted germination when the embryos are grown on media that emit no CO_2_ such as rockwool cubes. Elevating CO_2_ might facilitate the transition from heterotrophic embryos to autotrophic plants. That confirms the works of Kosai et al. (1997) who demonstrated the feasibility of photoautotrophic micropropagation using a sugar-free medium on different plant species. This method was applied to grow coffee plantlets from the interspecific hybrid *Coffea arabusta* from cotyledonary embryos in *in vitro* conditions. Cotyledonary-stage is the earliest stage capable of photosynthesizing due to a high chlorophyll contents (Afreen et al., 2002). To date, attempts to stimulate the transition to photo autotrophy in Arabica species using high concentrations of CO_2_ combined with high irradiation remain to be achieved. They could improve the rates of success in acclimation and conversion of embryos into seedlings.

#### Quality Control/System of Quality Assurance

In 2003, a quality assurance procedure was designed to guarantee the quality of the regenerated plants. Since this date, the mother plants have been regularly checked for the absence of *X. fastidiosa* using Elisa tests, since 2011 using micro-satellites according to Legendre et al. (2013). The absence of phytopathogenic microorganisms was also checked through regular and systematic axenic tests. The clonal identities of the tissues were checked by sampling the embryogenic callus and analyzing it using specific polymorphic microsatellites. Concerning the true-to-type quality of the regenerated plants, it is well known that the frequency of SV is related to the age of the *in vitro* cultures. Consequently, to limit this risk, it was decided to limit the period between the disinfection date of the leaves and the beginning of the production of an SE batch to 2 years. If auxin is used (IBA 5 mg L^-1^ or 2,4-D 1 mg L^-1^), its use is strictly limited to the first 5 months of the callus induction step. The multiplication in liquid medium is in all cases limited to 6 months.

### Technology Transfers for the Propagation of Selected Robusta Clones

In the mid-2000s, Nestlé decided to strengthen its green coffee supply chain with a global initiative launched in 2010: the Nescafé Plan^5^. For Robusta, the goals were to promote modern agricultural practices in producing countries as well as renovating old plantations with clones selected for yield, cup quality and disease resistance. The production potential of strategic markets was identified, including Mexico, Thailand, and the Philippines. To address the lack of a professional coffee plant propagation sector, the company invested in SE technology, not only to speed up the distribution of valuable Robusta plants. According to the countries, SE was either implemented in Nestlé R&D facilities (laboratory and nurseries) or locally transferred to public or private plant production units. When required, protocols (handbooks), laboratory or nursery layouts and training courses were provided.

#### Thailand

In 1998, the Department of Agriculture (DOA) and Nestlé Thailand decided to improve the yield of this crop by replacing the low yield trees with selected clones. The technique of SE was transferred to the DOA by means of a 1 year training of two scientists from the Chumphon Horticultural Research Centre (CHRC). The CHRC team produced and sold 0.55 M plants directly to the farmers and/or to Nestlé Thailand (Sanpote et al., 2006). A second propagation project started in 2005 with greenhouse facilities set up in the Nestlé’s factory. Between 2005 and 2010, 1.8 M somatic seedlings were distributed to the farmers (Kunasol et al., 2010).

#### Mexico

To fill the gap between the lack of coffee beans and increasing consumer demand, Nestlé decided to transfer the SE process to a local company and to implement it there to boost the propagation of Robusta plantlets, an attractive alternative in the context of severe attacks of leaf rust disease in the Arabica species. The technology was transferred from Nestlé Mexico, the Mexican institute of Agronomic Research INIFAP to NSIP (formerly Agromod SA de CV), a private company with well-known expertise in plant micropropagation.

Due to the allogamous feature of *C. canephora*, a set of four Robusta varieties (clones) was designed to start the mass propagation. The objectives of the technology transfer were to (i) insure reliable production of embryogenic callus on solid and in liquid medium, (ii) insure efficient differentiation of the embryogenic callus into somatic embryos, and (iii) implement an effective acclimation protocol that would allow a 50% embryo-to-plantlet conversion rate. Quality control was also implemented throughout the production process. In particular, the SE-derived plant root system was evaluated prior to distribution.

As a result, the process was fully transferred between 2012 and 2014 and adapted for efficient plantlet production (Breton et al., 2017). A laboratory with a production capacity of 10 M somatic embryos per year was fully operational in 2014. Dedicated nurseries were designed to achieve an embryo-to-plant conversion mean rate of 50%.

#### Philippines – A Case of Technical Assistance

**FIGURE 9:**
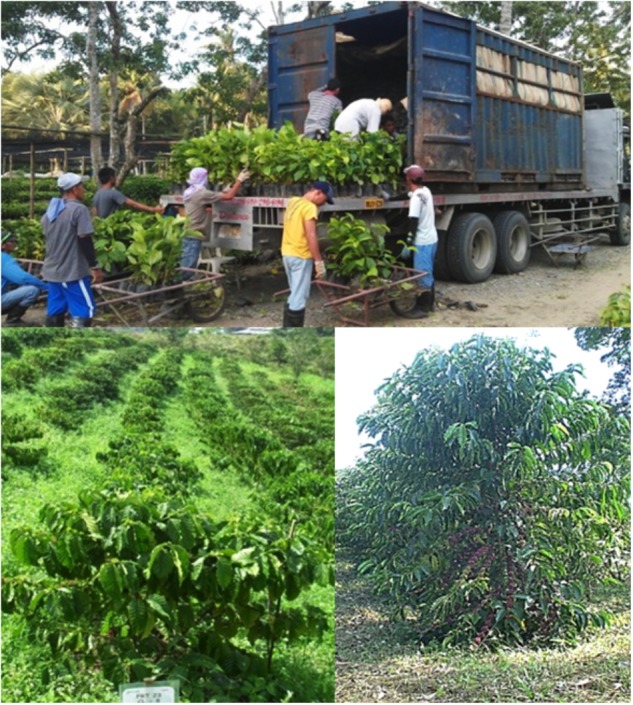
Robusta SE plants produced in Nestlé’s French facility and distributed to farmers in the Philippines **(Top)**; young trees in the field (Mindanao Island, **Bottom Left**); locally produced SE tree bearing fruits in a Mexican plantation (Chiapas, **Bottom Right**).

With the same objective, i.e., to be able to purchase coffee beans locally, Nestlé promoted the production and delivery of SE plantlets to farmers. This project has been underway since 2009. In their French laboratory, the Nestlé R&D team produces somatic embryos from existing embryogenic calli obtained from elite varieties using *in vitro* processes. The embryos are then pre-germinated in bioreactors to make them compatible with the greenhouse processes. Batches of embryos (around 1 M a year) are regularly shipped by plane from Tours to the city of Cabuyao, close to Manila. From there, they are transferred to Dalwangan (island of Mindanao) in the south of the archipelago. After this 5 day trip, the somatic embryos are sown, germinated, acclimated and grown until they reach the required size for transplantation in the field (Figure 9). Agronomists proceeded with the distribution of the coffee trees and provided technical assistance to farmers to ensure the plants express their full agronomic potential. Concomitantly, scientists from the Filipino Department of Agriculture were trained in the use of SE procedures at the Nestlé facility to promote the diffusion and use of the propagation technique. Between 2009 and 2017, around 1.9 M of Robusta SE trees were distributed to farmers (De Smet W., pers. comm.). This strategy enabled the start of mass propagation of elite clones of Robusta, juvenile SE plants being used as donor plants for classic rooted cutting techniques. Agronomists reported that a total number of 17 M trees were distributed thanks to this project.

#### Lessons Learned

From those technology transfers, lessons were learned mainly concerning the time required to master the acclimation step of SE plants. Managing such soft and fragile plants requires strict control of the climatic and phytosanitary environment. Also, logistics and plant transport require particular care, especially in countries where the nurseries are located in remote places. Communication with the farmers themselves is also required on how to grow young SE plants, especially when local farmers are used to working with seedlings. Moreover, despite the fact scientists from governmental institutions were trained, few of them actually implemented the SE production locally, and so the initial investment for the production unit remained prohibitive. Significant SE production was only maintained when a private company was involved, highlighting the crucial role of investors in the promotion of SE technology. Impacts were observed at the national scale, as illustrated in Mexico where 10.2 million Robusta SE-derived plants were distributed to farmers from 2010 to 2017. When all these trees are fully productive and assuming a yield of 2 tons per hectare, it can be expected that around 18.6 kilotons of green coffee will be harvested in Mexico, corresponding to around 40% of local demand (Garcia Alvarez O., pers. comm.).

## Somatic Embryogenesis, a Powerful Tool for Coffee Functional Genomics

### Genetic Transformation and Functional Genomics

Genetically modified coffee plants have been designed in the past 15 years by different research groups worldwide with three main objectives: to develop efficient transformation and regeneration methods, to decipher gene function and/or to improve coffee genotypes. However, as neither consumers nor coffee stakeholders want GMO coffee, CIRAD has explored genetic transformation and functional genomics as powerful tools to understand gene function.

**FIGURE 10:**
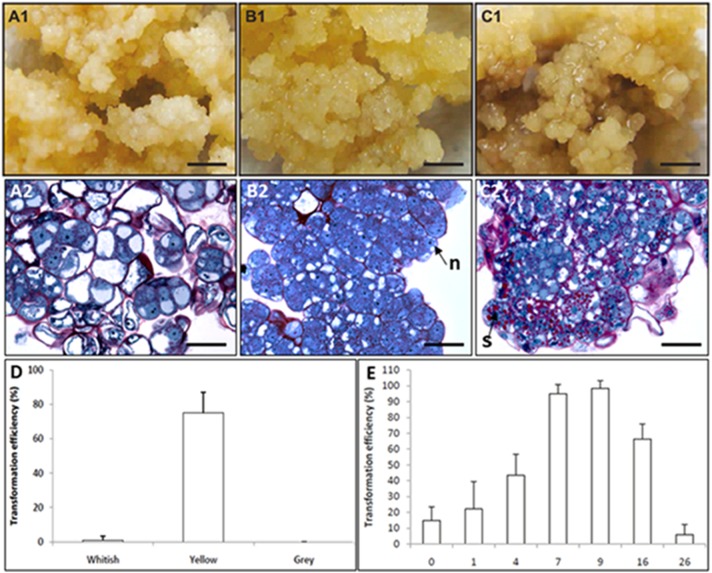
Coffee genetic transformation. Morphological **(A1,B1,C1)** and histological **(A2,B2,C2)** aspect of the different callus phenotypes observed in maintained embryogenic cultures assayed for coffee genetic transformation: **(A1,A2)** whitish type; **(B1,B2)** yellow type competent for genetic transformation comprising proembryogenic masses (PEMs); **(C1,C2)** gray type. **(D)** Transformation efficiency depending on the phenotype of maintained embryogenic callus cultures. **(E)** Effect of the age of the embryogenic callus culture on *A. tumefaciens*-mediated transformation efficiency (from Ribas et al., 2011).

Although direct and indirect DNA delivery systems have been successfully developed to transform coffee cells, most published studies used only reporter genes and described methodological improvements (Ribas et al., 2005b; Mishra and Slater, 2012). Despite these significant methodological advances in the transformation process itself, there are very few examples of the introduction of genes of agronomic interest in coffee, reflecting the difficulty faced by many laboratories to achieve regeneration of transgenic plants. Essential progress in establishing reliable and efficient transformation protocols in coffee has been achieved by optimizing the production conditions of embryogenic cultures used as target tissues for transformation. The availability of in depth SE knowledge and well mastered procedures played key roles in removing the remaining major bottlenecks. Hence, proembryogenic masses (PEMs) were shown to be the competent target tissue (Figure 10) and improved transformation efficiency is also systematically associated with increased quantities of PEMs depending on the adequate auxin/cytokinin balance, number of preliminary proliferation cycles on auxin rich medium and low mineral salt strength (Ribas et al., 2011).

Some of the genes isolated from coffee were analyzed using a functional genomic approach comprising a theobromine synthase gene (*CaMXMTI*) to suppress caffeine biosynthesis (Ogita et al., 2003, 2004), an *ACC oxidase* gene involved in ethylene biosynthesis (Ribas et al., 2005a) and a *CaHSFA9* and *CaDREB2* gene respectively involved in the transcriptional activation of heat shock proteins (HSPs) and tolerance to heat and drought stress (Dussert et al., 2018). The promoter of *CcDREB1B* (Alves et al., 2017) and *CaWRKY1a* and *CaWRKY1b* (Petitot et al., 2013) were analyzed in transgenic coffee plants. A few studies conducted by other teams aimed to improve coffee genotypes with desirable traits (Leroy et al., 2000; Cruz et al., 2004; Ribas et al., 2005a, 2006; Arroyo-Herrera et al., 2008).

### Genome Editing by CRISPR/Cas9

On July 2018, the Court of Justice of the European Union (ECJ) in Luxembourg ruled that gene-edited crops should be subject to the same stringent regulations as conventional genetically modified (GM) organisms (2001 directive)^6^. Moreover, organisms made using mutagenesis techniques developed after 2001 are not exempt from the 2001 directive relative to GMO regulation. This very rigid judgment goes against legislations in other countries such as the United States where the law exempts organisms whose genomes were modified using mutagenesis techniques, such as irradiation or genome editing, which introduce changes to an organism’s DNA but does not add foreign genetic material. The coffee sector does not want GMO, but how is it going to consider the edited plants?

Genome editing technologies, which are an unprecedented technological breakthrough, provide novel opportunities for functional genomic studies and molecular breeding but rely on highly efficient genetic transformation and plant regeneration methods, which could be bottlenecks in the process (Altpeter et al., 2016; Buell and Voytas, 2017). One of them, the CRISPR/Cas9 technology is simple, versatile and cheap and provides a valuable means of creating targeted mutations and sequence replacements in metazoan and plant genomes with high specificity (Ding et al., 2016; Quétier, 2016). Although reports of genome editing in tree species are still limited (Jia and Wang, 2014; Fan et al., 2015; Zhou et al., 2015; Nishitani et al., 2016; Wang et al., 2018), this technology was recently successfully used to introduce mutations in the coffee genome (Breitler et al., 2018). Nevertheless, the use of SE as the method for regenerating genome-edited plants could restrict the choice of targeted genes to those that are not essential to the somatic embryo development and germination steps. For other genes, an alternative is to set up for coffee the shoot regeneration method used for other woody species. Indeed, poplar and apple tree albino plants were obtained using the same method of genetic transformation and further regeneration. Leaf disks were dipped in *Agrobacterium* culture and cultured first on callus-induction medium and then on screening medium, to induce adventitious buds and finally shoot formation (Fan et al., 2015; Nishitani et al., 2016).

The recent advances in coffee genomics, including the improvement of coffee genetic transformation techniques and genome editing (Alpizar et al., 2008; Ribas et al., 2011; Déchamp et al., 2015; Breitler et al., 2018), now open the way for studies of the molecular and genetic determinism of many important agronomic traits, leading to the identification of molecular markers and candidate genes that can now be routinely functionally validated thanks to efficient genetic transformation pipelines, use of cryopreserved competent embryogenic calli and well established bioassay conditions (Marraccini et al., 2011, 2012; Vieira et al., 2013; Mofatto et al., 2016).

## Current Researches on Coffee Somatic Embryogenesis

### Unraveling the Molecular Mechanisms Underlying the SE Process

As discussed above, research on SE has led to the successful industrialization of the process for the large-scale dissemination of improved coffee varieties of both *C. arabica* and *C. canephora* species. Over the last 12 years, intensive research has enabled the design of mastered and reproducible SE protocols, well-suited for all commercially propagated genotypes at acceptable production costs.

However, like for the other crops, coffee SE research remains mainly empirical, characterized by a low-throughput and hypothesis-driven research, leading to an overall slow technical progress. For example, the development of culture conditions for the establishment at the industrial level of embryogenic cell suspensions in *C. arabica* and the mass regeneration of somatic embryos have been very long (10 years) and laborious, carried out only by an empirical approach. The lack of knowledge about the cellular and molecular events taking place during these developmental stages makes these stages real black boxes. While a scale-up is needed to meet increasing market demand – estimated at 50–100 million vitroplants per year – current overall production cannot meet this huge demand, mainly due to the lack of efficiency of certain steps in the SE process. We believe that the lack of understanding of the molecular mechanisms underlying the reprogramming of somatic cells is the main limitation to improving coffee SE protocols.

Recently, Campos et al. (2017) provided an overview of which achievements and molecular insights have been gained in coffee SE and encourage researchers to invest further in the *in vitro* technology and combine it with the latest omics techniques. Since the last 16 years, several works have evidenced the potential of omics technologies to optimize coffee SE. For example, Silva et al. (2015) showed higher expression levels of two BBM homologous sequences in embryogenic calli and cell suspensions of *C. arabica* when compared to non-embryogenic calli. They also identified SERK orthologs in embryogenic cell suspensions (Silva et al., 2014). Arroyo-Herrera et al. (2008) showed that an overexpression of a heterologous gene of WUS led to a huge increase in the production of somatic embryos. Quiroz-Figueroa et al. (2002) evidenced differential gene expression in embryogenic and non-embryogenic clusters from cell suspensions in *C. arabica*. Tonietto et al. (2012) reported the proteomic analysis of developing somatic embryos of *C. arabica* leading to the identification of 14 proteins linked to different developmental stages during SE as enolase and globulin S11 for torpedo stage. The proteome profile of embryogenic calli was recently published (Campos et al., 2016). It was shown that epigenetic regulation of genes could play a role in coffee SE. Nic-Can et al. (2013) proved that the genes LEC1, BBM1, and WUS related gene WOX4 are under epigenetic control in relation to the embryogenic capacity in *C. canephora*. However, these studies were carried out on several different protocols, in research laboratories and using non-optimized and often asynchronous SE regeneration processes giving rise to the co-existence of several stages of development in a same sample.

Today, a reliable, synchronized and efficient industrialized SE process, added to the recent boom in -omics technologies represents a real opportunity to understand the molecular events involved in the key developmental switches of coffee SE and those associated with the main phase changes, for example, at the transcriptomic (gene expression) level, associated with totipotency.

To better understand SE, it is important to look at the process as a sequence of events or developmental stages. These stages were chosen and characterized based on well described morphological and anatomical changes that occur when switching from one stage to another (Jayasankar et al., 2003). Nine developmental stages have been characterized in the indirect SE of dicots: explant, primary callus, embryogenic callus, PEMs, globular, heart-shaped, torpedo-shaped and cotyledonary embryos, and plantlet (Figure 1). During direct SE, globular embryos appear directly on the explant with no intermediate callus phase and as this process is generally asynchronous it is not suitable for this kind of basic study in which the synchronous regeneration of well identified developmental stages is a prerequisite.

To undertake a comprehensive analysis of the indirect SE process in *C. arabica*, CIRAD and Nestlé R&D launched a significant joint research project, firstly to study the transcriptional changes that occur at the key developmental switches. With the very shortly expected delivery of the *C. arabica* genome sequence (Mueller et al., 2015), applying combined NGS techniques will make it possible to create a gene expression profile blueprint of coffee SE. The availability of the *C. arabica* genome follows the publication of the *C. canephora* genome (Denoeud et al., 2014).

In parallel to transcriptomic and detailed phenotyping approaches, full metabolomic profiles and the hormone contents at each of the developmental stages are being drawn. Visualizing the evolution of the primary and secondary metabolism at each stage and its interaction with hormone content and levels will help characterize cell functioning at each stage.

Integrating these results in the transcriptomic profiles obtained will definitely help to build the first regulatory networks of the Arabica SE process as a whole – an illustration of totipotency. As well as the huge basic knowledge accumulated, these networks will subsequently help identify candidate molecular and biochemical markers known to be key factors in the success of the different developmental stages. These markers will then enable further optimizations of the SE process (Campos et al., 2017), ending 40 years of empirical research.

### High-Throughput Screening of Active Compounds to Optimize SE

**FIGURE 11:**
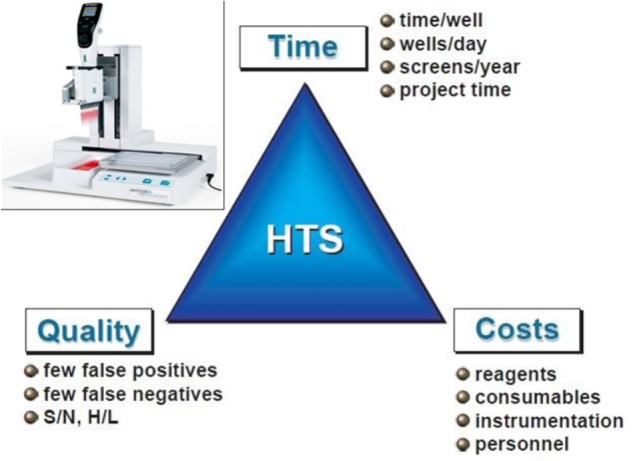
The keys to successful high-throughput screening (HTS). Optimization of screening systems is evaluated according to 3 criteria: time, cost, and quality. S/N, signal-to-noise ratio; H/L, High-to-low ratio (sensitivity of the screening; *adapted from Mayr and Fuerst, 2008)*.

Testing the effect of new compounds on SE efficiency is generally a laborious, intensive and tricky operation, which prevents considering comparing the effects of a large number of molecules and by that, greatly prevents accelerating culture media optimization. Nestlé R&D is working on a high-throughput screening (HTS) of active compounds to optimize SE protocols. A proof of concept of the high-throughput approach has been successfully validated in coffee with a first set of molecules and already allows the multiplication of experimental conditions, reduces culture volumes by a factor of a hundred as well as the space and labor required compared to the conventional research approach used until now. The next step is to scale up this technology through the screening of chemical libraries on robotic platforms. Figure 11 shows the key factors in the success of HTS (Mayr and Fuerst, 2008). Once molecular knowledge about the SE process is gained thanks to the cutting edge -omics technologies described above, target-based active compound screening can be applied on a reasoned and scientific basis to optimize SE protocols. Adding active compounds to the culture medium, that have a potential effect on SE targets, and are capable of modulating the physiological state of cells, is a possible way to maximize embryo production, quality, and remove genetic (genotypic effects) or stress-induced recalcitrance. To effectively select active substances, it is now possible to screen hundreds of libraries (Rodriguez-Furlan et al., 2016). Nestlé is currently reproducing some SE phases at a miniaturized level compatible with an automated screening platform, which appears to be unavoidable to reach the high-throughput standards.

## Conclusion: Tomorrow’s Challenges for Large-Scale Coffee Dissemination by Somatic Embryogenesis

Coffee SE is now one of the most advanced technologies. It has been industrialized (Robusta) or commercialized (Arabica) for 13–15 years for the two cultivated species. Very high yields and biological efficiency characterize both processes, with low genotypic effects and controlled SV. To date, very few examples of commercial applications of SE are available for any species. The industrial application of SE in hardwoods remains limited to a few species such as the hybrid yellow-poplar, cocoa, and coffee. Thirty five M improved coffee clones derived from SE have already been planted in the field.

Research by the two leading groups – the CIRAD/ECOM alliance and Nestlé – has often been way ahead and the resulting innovations have benefited the entire SE working community. This is particularly true for the temporary immersion bioreactors that are now extensively used for the micropropagation of many different species. It is interesting to observe the complementarities of the research done by these two teams and how they have facilitated the rapid development of micropropagation of both Arabica and Robusta.

The objective of the Nescafé Plan is to reach a total of 220 M coffee plants (Arabica and Robusta) distributed to farmers worldwide by 2020, using any method of propagation (seedling, rooted cuttings from SE-derived plants, grafting and SE). The price of Robusta plants derived from SE was a limiting factor in the Philippines, which explains why rooted cuttings from SE-derived plants was privileged. Today, a total of c.a. 15 M of Robusta plants are reported to have been distributed to farmers using the SE method. To our knowledge, this is one of the biggest commercial SE-based propagation achieved in woody plants so far, however, it is still far behind the 74.5 M SE cocoa trees produced by ICCRI (Indonesian Coffee and Cocoa Research Institute) between 2009 and 2011 (Maximova and Guiltinan, 2015). The ECOM/CIRAD alliance has evaluated the demand for Arabica hybrid vitroplants at several dozen million per year for the next 5 years. The interest of these hybrid clones for agroforestry systems, to whose environmental conditions they are particularly well suited because of their high photosynthetic efficiency under low light, is boosting demand.

Based on the previous situations described for Thailand, Nicaragua, Costa Rica, Mexico, and the Philippines, SE is now a must-have in the toolbox of the Arabica and Robusta coffee plant producers. This *in vitro* method, already validated on more than 20 Robusta clones and 40 Arabica F1 varieties, allows significant time and space savings, and offers greater versatility for tree propagation. Indeed, breeding programs are now delivering new varieties (i.e., with improved cup quality as well as new disease resistant or drought tolerant clones) faster that can be rapidly deployed in the field. The SE process can also supply axenic planting material, which may be a prerequisite for successful international shipments. In a context of increasing parasite pressure including the major risk of the spread of *X. fastidiosa* or soil nematodes (*Meloidogyne* or *Pratylenchus* sp.), micropropagation has the great advantage of guaranteeing plants that are healthy and free from pests and diseases. This advantage will become increasingly important in the future.

Obviously, this system of propagation requires significant investments (laboratory and nursery) and skilled technicians to be effective. Training staff, who may need a long time to acquire sufficient experience, also has to be taken into account. Further improvements, especially focused on the automation of the acclimation step *ex vitro* are expected to dramatically reduce labor costs due to the need to handle each individual plant. In another connection, new technologies have recently been developed by the plant micropropagation industry for cost savings in the laboratory (LED lights, CO_2_-enrichment, etc.). These developments may result in reconsidering the original strategies, particularly by investigating how embryo sowing could be automatized in the laboratory, using for example the Vitro Plus process implemented for the production of fern vitroplants^7^.

Contrary to what might have been expected a few years ago, SV has not been a bottleneck for the SE development for both cultivated coffee species. For each cultivated coffee species, a limited genotypic effect allows at the industrial scale the utilization of a common protocol whatever the cultivar propagated. The costs of production are more limiting. The use of *in vitro* cultivation to propagate new selected varieties represents in all cases an additional cost compared to traditional varieties that are agronomically less efficient but distributed at low cost in the form of seedlings, sometimes free of charge by governments. In spite of the very low selling prices reached with coffee vitroplants, they are often still too high for small farmers, but acceptable for larger producers who are aware of the agronomic added value. Line losses during the acclimatization and nursery stages (50% losses) are costly and significantly increase production costs. This problem is more and more minimized by the generalization of the use of mini-cuttings from SE-derived plants with which the losses are almost zero during these stages and hence the industrialization possible.

The coffee community working on SE chose to conduct in parallel the following strategies: to increase our knowledge about the molecular mechanisms controlling SE, to optimize SE by using innovative methods based on piloting with molecular markers and screening of active compounds using a miniaturized and automated system, to automate the acclimation step, to industrialize and generalize the rooted mini-cuttings method from somatic seedlings, to boost the market for Arabica hybrids and Robusta clones by bringing roasters and farmers closer together around innovation platforms so that the added value of improved varieties is better known, to democratize methods of cloning coffee and attract large micropropagation companies with the double aim of scaling up the coffee vitroplant production and being present in a maximum of producing countries.

This paper clearly shows that the success of coffee SE is tightly linked to the availability of selected varieties and clones to propagate. Up to now, SE research and development has been able to support the dissemination of new clones. However, a new huge up scaling appears to be necessary to be able to rapidly provide much larger quantities. In the case of Arabica, the reason is the recent loss of leaf rust resistance in all the cultivated conventional resistant varieties and the increasing impact of the harmful effects of climate change. In the case of Robusta, there is also an urgent need to replace unselected clones with improved ones, particularly in Vietnam – the world’s main coffee producer – where coffee orchards need to be totally renewed.

The absence of a functioning seed industry is a major deterrent for perennial tropical crops in many developing countries. Given the long lifespan of a tree plantation and hence the low renewal rate compared with annual crops, the propagation of perennial species has not been attractive so far as it is deemed uneconomic by investors. The investment of large micropropagation companies in the coffee planting business would guarantee the success of this future change of scale. With the availability of new improved clones and their economic added value, relevant business cases for the production of coffee plants should be disseminated to draw the attention of investors to the use of SE. All past and present efforts toward the democratization of coffee SE should generate huge interest in new varieties and the associated propagation techniques.

## Author Contributions

HE organized the plan and wrote the whole manuscript. DB and J-PD wrote all the parts concerning the Robusta SE. J-CB, SL, ED, CC, and RA wrote the current research part. BB, PM, EA, and HE were in charge of the analysis of social and economic constraints affecting *C. arabica*. EA, HE, FG, and PC wrote the paragraphs related to Arabica SE professionalization, technology transfer, and rooted mini-cuttings.

## Conflict of Interest Statement

The authors declare that the research was conducted in the absence of any commercial or financial relationships that could be construed as a potential conflict of interest.

## Footnotes

1 http://www.breedcafs.eu/

2 http://www.agdia-biofords.com/en/prod

3 https://worldcoffeeresearch.org/work/seed-and-nursery-verification-program/

4 https://worldcoffeeresearch.org/work/variety-intelligence

5 https://www.nestle.com/csv/raw-materials/coffee

6 https://www.nature.com/articles/d41586-018-05814-6

7 http://www.vivi.nu/
